# Multimodal large language models for bladder tumor detection in cystoscopy: a retrospective benchmarking study

**DOI:** 10.1007/s00345-026-06761-y

**Published:** 2026-09-17

**Authors:** Yonatan Prat, Husny Mahmud, Abraham Tsur, Menachem Laufer, Dina Orkin, Zohar A. Dotan, Barak Rosenzweig

**Affiliations:** 1 Department of Urology, Sheba Medical Center, Ramat Gan, Israel; 2 ARC Innovation Center, Sheba Medical Center, Ramat Gan, Israel; 3 Gray Faculty of Medical and Health Sciences, Tel-Aviv University, Tel-Aviv, Israel; 4 The Dina Recanati School of Medicine Reichman University, Herzliya, Israel; 5 The Josef Buchmann Gynecology and Maternity Center, Sheba Medical Center, Ramat Gan, Israel; 6 Department of Anesthesia, Sheba Medical Center, Ramat Gan, Israel; 7 The Talpiot Medical Leadership Program, Sheba Medical Center, Ramat Gan, Israel

**Keywords:** Bladder cancer, Cystoscopy, Artificial intelligence, Large language models, Prompt engineering

## Abstract

**Purpose:**

Cystoscopic assessment is central to bladder cancer diagnosis, yet visual interpretation remains variable. Existing artificial intelligence approaches often depend on data-intensive models that are often difficult to deploy in routine practice. We evaluated whether multimodal large language models (MLLMs), including smaller and more efficient architectures, can accurately classify cystoscopy images, and whether prompt engineering improves performance. The primary outcome was benign-versus-malignant classification. Secondary outcomes included calibration, high-confidence triage, and performance stratified by imaging modality.

**Methods:**

We retrospectively analyzed 1,754 labeled public cystoscopy images. Three prompt types were tested: Direct, Book-based, and Optimized, across GPT-5.2, GPT-5, GPT-5-Mini, and GPT-5-Nano. Performance measured: accuracy, sensitivity, specificity, and F1 Score. Confidence evaluation: using Brier Score and Expected Calibration Error. High-confidence triage using abstention option based on loss function.

**Results:**

GPT-5 and GPT-5-Mini with the optimized prompt achieved the best benign-versus-malignant performance, with accuracies of 86.7% and 89.2%, specificities of 94.1% and 88.4%, and sensitivities of 82.5% and 89.2%, respectively. GPT-5 with the optimized prompt achieved the best high-confidence triage performance, yielding 98.1% accuracy, 94.6% specificity, and 99.1% sensitivity at 62.0% image coverage. Prompt engineering improved model performance, although these gains were not statistically significant, and enhanced confidence calibration and triage performance.

**Conclusions:**

This retrospective evaluation demonstrates the potential of MLLMs for cystoscopic bladder lesion classification. Prompt engineering improved diagnostic calibration and output reliability, while high-confidence triage increased accuracy to 98.1%, supporting the feasibility of MLLMs as foundation models for cystoscopic assessment.

**Supplementary Information:**

The online version contains supplementary material available at https://doi.org/10.1007/s00345-026-06761-y.

## Introduction

Bladder cancer (BC) is the 10th most prevalent malignancy globally and the sixth most common cancer in men [1]. Approximately 75% of cases present as non–muscle-invasive bladder cancer (NMIBC), a disease subtype with high recurrence that necessitates intensive, long-term endoscopic surveillance [2]. In the management of BC, cystoscopy serves as the cornerstone for primary diagnosis, therapeutic intervention, and follow-up, a status reflected in its central role within major clinical practice guidelines [3–5]. Within cystoscopy, modality differences further complicate standardization. While enhanced imaging such as Narrow-band imaging (NBI) can improve detection of certain lesions, practical barriers limit ubiquitous use [5–7]. White-light imaging (WLI) remains the default in many clinics, with known miss rates of 10% to 30% for bladder tumors a diagnostic gap that contributes directly to incomplete resections and, consequently, high rates of early recurrence [8, 9]. In this mixed-modality reality, systems that can harmonize decision criteria across WLI and NBI are particularly valuable [8]. Over the past decade, computer vision systems based on artificial intelligence (AI), have sought to improve and standardize detection and reduce observer variability of cystoscopy [10]. Across multiple reports, including multicenter studies, these systems have achieved high diagnostic accuracy, sensitivity, and specificity, at times rivaling expert performance [2, 11]. Nonetheless, AI development has depended on large, carefully curated, labeled datasets and substantial engineering effort, creating barriers to rapid iteration, domain transfer and standardization [12–15]. A newer paradigm, multimodal large language models (MLLMs) offers a different path [16]. By jointly processing multiple inputs such as images, and text, MLLMs can work as a foundation model for cystoscopy management [16, 17].

Despite early feasibility reports, evidence is limited regarding the reliability of MLLMs for cystoscopic image classification under real-world constraints and mixed WLI/NBI use [17]. Moreover, the current literature shows that prompt engineering (PE) plays a key role in large language models (LLMs) performance [18–20]. However, the influence of PE; definitions, class boundaries, and contextual instructions on performance and especially calibrationhas not been systematically evaluated in cystoscopy. In our study we accessed MLLMs with PE. It represents a timely opportunity to improve reliability, robustness and consistency of cystoscopic decision support [14, 21]. Accordingly, we aimed to quantify the impact of PE on performance by: (1) testing whether MLLMs accurately classify cystoscopic images; (2) assessing model confidence and calibration; (3) evaluating reliability using high-confidence triage strategy; and (4) conducting modality-stratified analyses [22, 23].

## Materials and methods

### Study design and overview

We conducted a retrospective head-to-head evaluation of four general-purpose MLLM (GPT5.2,GPT5, GPT5-Mini, GPT5-Nano) to perform classification of cystoscopy images under three prompting strategies: (**P-1**) direct prompt, (**P-2**) Book-Based Prompt, and (**P-3**) Optimized Prompt (Fig.SI1).

### Dataset

A public dataset comprised of 1754 labeled cystoscopy images from 23 patients undergoing Transurethral Resection of Bladder Tumor (TURBT) was used [24]. Images were reviewed by expert urologists and then labelled with pathological information from the resected lesions [25]. Ground-truth labels followed WHO/ISUP conventions used in bladder cancer literature. Two categories were considered malignant: Low Grade and High Grade (LG U HG). Two categories were considered benign: No-Tumor-Lesion and Non-Suspicious Tissue, NTL U NST [24]. Images included both WLI and NBI modalities (WLI = 1433, NBI = 321)(Table.SI1).

### AI model and prompting strategies

OpenAI’s models: GPT5.2,GPT5, GPT5-Mini, GPT5-Nano are capable of analyzing both textual and image inputs simultaneously. All models were accessed using Python 3.9+. Each image underwent 12 independent inference conditions (4 models × 3 prompts), yielding 21,048 total image classification (12 × 1,754). Each image produced a four-class probability vector over NST, NTL, LG and HG, constrained by explicit JSON output schema to sum to 100% [26]. Each model was evaluated under three prompt configurations representing increasing levels of prompt-engineering complexity.

1. Direct prompt (P-1)
  The model was instructed to assume the role of a urologist and return a probability distribution over HG, LG, NTL, and NST. No reasoning guidance was provided and no examples given (zero-shot). This serves as a baseline direct query.
2. Book-based prompt (P-2)
  P-2 augmented P-1 with domain knowledge by appending succinct, textbook diagnostic descriptors for each class- HG, LG, NTL, and NST- adapted from Campbell-Walsh-Wein Urology, 13th edition.
3. Optimized prompt (P-3)
  P-3 further refines P-2 using established prompt-engineering methods: it specifies explicit decision rules, permits internal self-reflection/backtracking, and applies tiebreakers for near-equivocal outputs. The instructions also direct the model to ignore non-tissue artifacts (e.g., endoscopic instruments), focus on most suspicious tissue and include guardrails that favor conservative decisions to reduce false negatives [19, 27]. This technique is intended to let the model improve accuracy but even more importantly, improve confidence and calibration.

## Outcomes measures

### Primary outcome

The primary outcome was diagnostic performance for each method (prompts and models) on Benign vs. malignant classification. (Malignant = HG ∪ LG; Benign = NST ∪ NTL).

### Secondary outcomes

1. **Confidence evaluation**: Using Brier score (BRS) *and Expected calibration error (ECE)*.
2. **High-confidence triage**: Performance re-estimated on subsets of predictions exceeding a prespecified confidence threshold.
3. **Modality stratification**: WBI vs. NBI diagnostic performance for each method.

## Statistical analysis

### Diagnostic performance

Analyses were performed per image. For the binary task, malignant probability was $\:{p}_{\mathrm{Mal}}={p}_{\mathrm{HG}}+{p}_{\mathrm{LG}}$and benign probability was $\:{{p}_{\mathrm{Ben}}=p}_{\mathrm{NST}}+{p}_{\mathrm{NTL}}$, and the predicted label was the class with the highest predicted probability. We computed accuracy, sensitivity, specificity, positive predictive value, negative predictive value, and F1-score (F1). For Confidence intervals (CIs) proportions we used Wilson score of 95% CI.

### Model confidence assessment

For an AI system to be reliable, the confidence (i.e. probabilities) it expresses in its decisions must match its accuracy. In this study we report two scores on for each model uncalibrated probability outputs: (1) *BRS*: the mean squared error between confidence and the binary true outcome (correct classification vs. incorrect classification) across all cases [28, 29]. (2) *ECE*: the weighted average difference between confidence and accuracy in discretized probability bins, with lower ECE indicating better alignment between predicted confidence and observed accuracy [29–31].

### High-confidence triage (abstention)

For high-confidence triage we did not retrain or alter the classifier. Instead, we applied a post-hoc selective classification layer (reject option) on top of the frozen outputs [14, 21, 23, 32]. A sample was auto-classified as: (1) Malignant, when the probability of malignant ($\:{p}_{\mathrm{Mal}})\:$was greater or equal to a threshold ($\:{p}_{\mathrm{Mal}}\ge\:{\tau\:}_{\mathrm{M}\mathrm{a}\mathrm{l}}$), (2) Benign when $\:{p}_{\mathrm{Mal}}\le\:{\tau\:}_{\mathrm{B}\mathrm{e}\mathrm{n}}$ and (3) abstained otherwise ($\:{\tau\:}_{\mathrm{B}\mathrm{e}\mathrm{n}}<{p}_{\mathrm{Mal}}<{\tau\:}_{\mathrm{M}\mathrm{a}\mathrm{l}}$) (Fig.SI2). Abstained cases are intended to be deferred to standard-of-care human review, thereby trading reduced automated coverage for higher reliability on the retained predictions. Thresholds selection was done via loss function minimization.

### Threshold selection

To select thresholds $\:\left({\tau\:}_{\mathrm{M}\mathrm{a}\mathrm{l}},{\tau\:}_{\mathrm{B}\mathrm{e}\mathrm{n}}\right)$, we optimized a cost-sensitive loss function designed to reflect asymmetric clinical risk [33]. False negatives (FN) were penalized more than false positives (FP), true positive (TP) and true negative(TN) were cost set to 0, while abstention carried a small penalty to discourage trivial “abstain-all” solutions [33]. On a held-out validation set split comprising 20% **(**287/1433 of images**)**, we computed loss function *(*$\:\mathcal{L})$ as follows:

$$\:\mathcal{L}({\tau\:}_{\mathrm{M}\mathrm{a}\mathrm{l}},{\tau\:}_{\mathrm{B}\mathrm{e}\mathrm{n}})=2.00\cdot\:\mathrm{F}\mathrm{N}+1.00\cdot\:\mathrm{F}\mathrm{P}+0.15\cdot\:\mathrm{A}\mathrm{b}\mathrm{s}\mathrm{t}\mathrm{a}\mathrm{i}\mathrm{n}+0\cdot\:\mathrm{T}\mathrm{N}+0\cdot\:\mathrm{T}\mathrm{P}$$

**Fig. 1:**
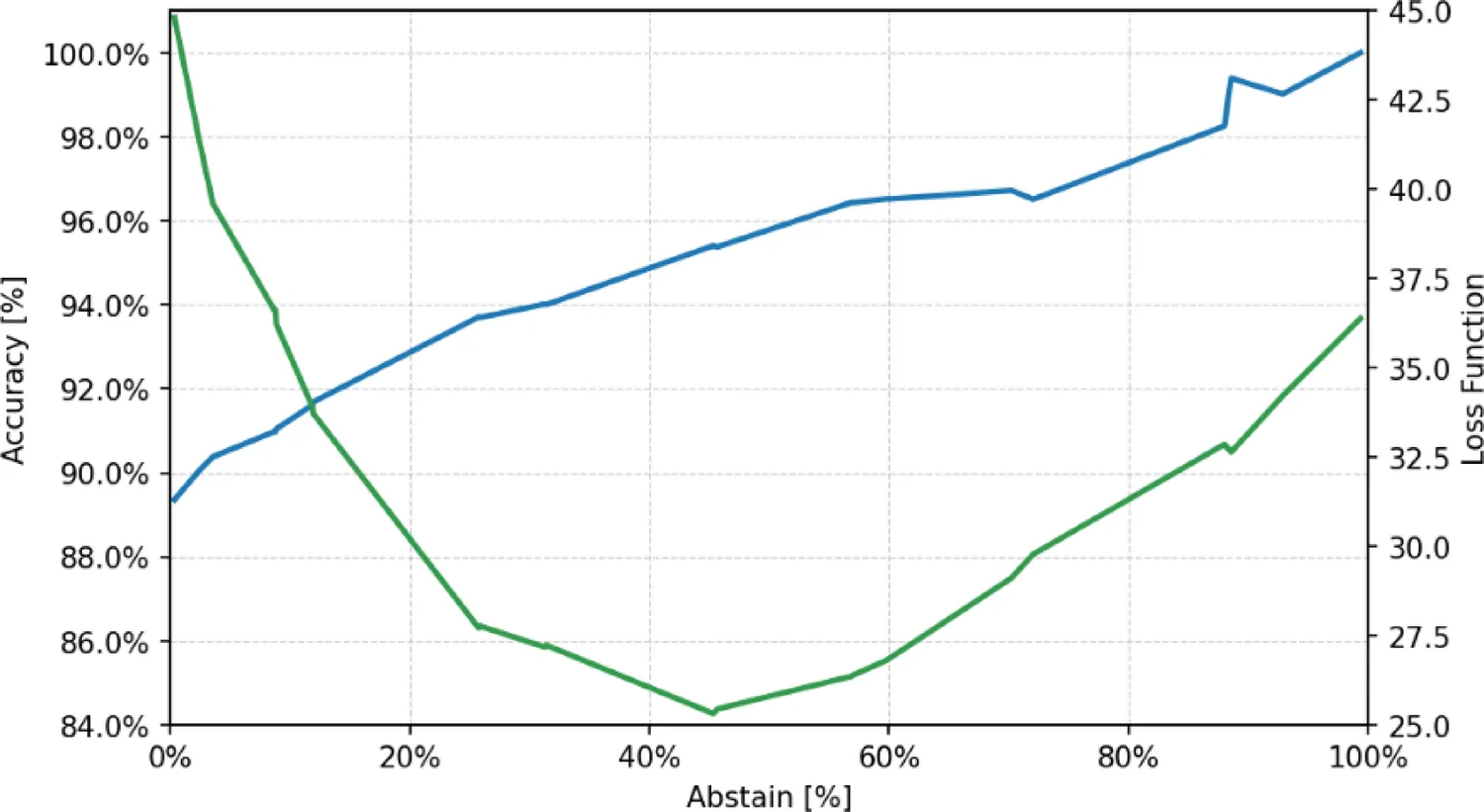
Accuracy and Loss Function vs. Abstention Rate. Accuracy (blue) increased with increasing abstention, approaching 100% at high abstention levels. The loss function (green) reached its minimum at an intermediate abstention rate, indicating the optimal trade-off between accuracy and model utility

We performed a discrete sweep over two-sided threshold pairs starting from $\:{\tau\:}_{\mathrm{M}\mathrm{a}\mathrm{l}}=0.50\:\:\:$and $\:{\tau\:}_{\mathrm{B}\mathrm{e}\mathrm{n}}=0.20$, updating thresholds in 1% increments (± 0.01) over the ranges $\:{\tau\:}_{\mathrm{M}\mathrm{a}\mathrm{l}}\in\:\left[\mathrm{0.50,1.00}\right]\:\:$and $\:{\tau\:}_{\mathrm{B}\mathrm{e}\mathrm{n}}\in\:\left[\mathrm{0.00,0.20}\right].\:$We recorded a loss function, $\:\mathcal{L}\left({\tau\:}_{\mathrm{M}\mathrm{a}\mathrm{l}},{\tau\:}_{\mathrm{B}\mathrm{e}\mathrm{n}}\right),\:$for each candidate pair and selected the thresholds that minimized $\:\mathcal{L}$ (Fig.SI3). This procedure yields a model-prompt specific operating point, for example $\:{\tau\:}_{{\mathrm{M}\mathrm{a}\mathrm{l}}_{\mathrm{G}\mathrm{P}\mathrm{T}5\_\mathrm{P}3}}=85\%,{\tau\:}_{{\mathrm{B}\mathrm{e}\mathrm{n}}_{\mathrm{G}\mathrm{P}\mathrm{T}5\_\mathrm{P}3}}=5\%$, that explicitly balances missed malignancies, unnecessary malignant calls, and the fraction of cases deferred for review. Using these thresholds, we calculated diagnostic performance, and coverage percentage (CVG) = 100% minus abstain%. Threshold-response curves were summarized to characterize the accuracy–abstention trade-off using one-side threshold (Fig. 1).

## Results

### Overall model performance WLI (malignant vs benign)

The diagnostic Performance was highest for GPT-5 with P-3 with 86.7% accuracy (95% CI, 84.9–88.4), 94.1% specificity (95% CI, 91.8–95.8), 82.5% sensitivity (95% CI, 79.9–84.8), and an F1 score of 88.7%. GPT-5-Mini achieved 89.2% accuracy (95% CI, 87.5–90.7), 88.4% specificity (95% CI, 85.4–90.9), 89.6% sensitivity (95% CI, 87.5–91.5), and an F1 score of 91.3% (Table SI2). Compared with P-1, P-3 increased accuracy by 1.0, 1.2, 0.4, and 5.0% points for GPT-5.2, GPT-5, GPT-5-Mini, and GPT-5-Nano, respectively. However, only the improvement for GPT-5-Nano remained significant (*P* < 0.001). Compared with P-2, only GPT-5.2 showed a significant improvement with P-3 (*P* = 0.006; Table. SI2b).

**Fig. 2:**
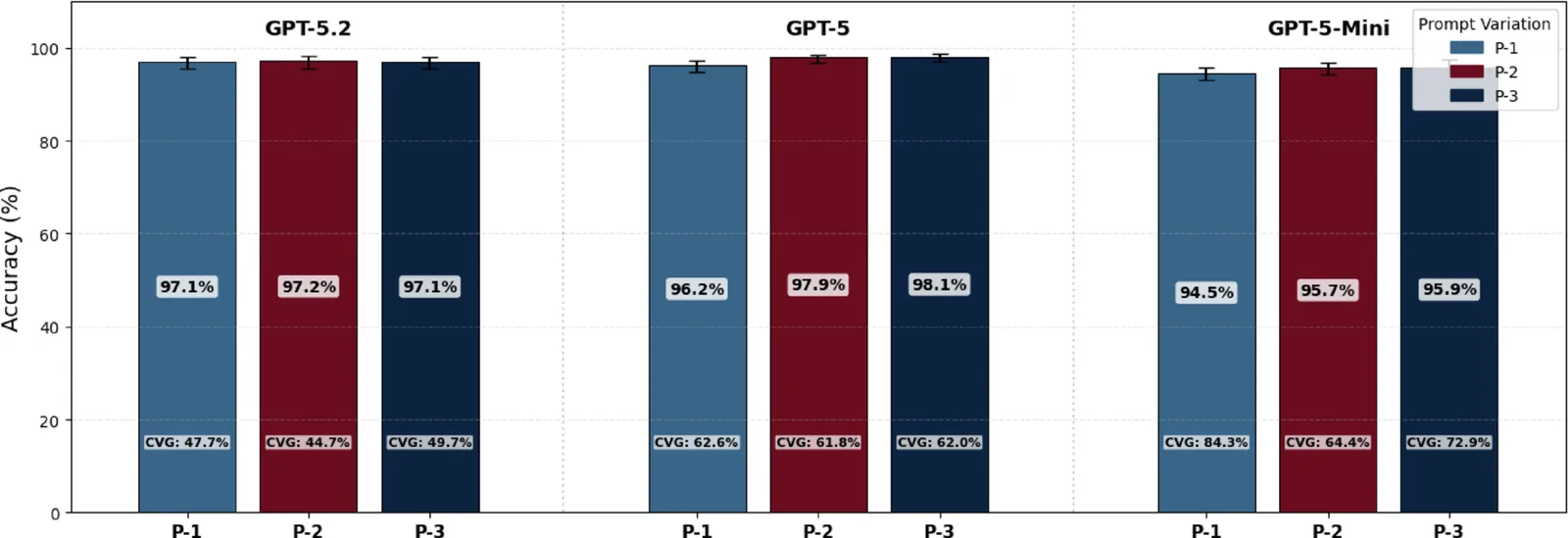
Accuracy by model × prompt classification of WLI images using High-confidence Classification. With high-confidence classification, all models achieved high WLI accuracy, ranging from 94.5% to 98.1%. GPT-5 with P3 showed the best performance, while prompt-related differences were modest across models. CVG varied between models, indicating different levels of case retention after abstention. *GPT-5- Nano is missing because the loss function favored the trivial solution, to abstain all images, for any $\:{\tau\:}_{\mathrm{M}\mathrm{a}\mathrm{l}},\:{\tau\:}_{Ben}$ CVG - Coverage of images from dataset

1. Model confidence assessment:
  The reliability of predicted class probabilities for malignant versus benign images depended on both the model and the prompt. P-3 yielded the lowest Brier score (BRS) across all models. Compared with P-2, BRS was significantly lower with P-3 for GPT-5.2, GPT-5, GPT-5-Mini, and GPT-5-Nano (all *P* ≤ 0.005). Compared with P-1, the reduction was significant for GPT-5.2 and GPT-5 (both *P* < 0.001), but not for GPT-5-Mini (*P* = 0.077) or GPT-5-Nano (*P* = 0.572). P-3 also produced the lowest expected calibration error (ECE), with values of 0.194 for GPT-5.2, 0.092 for GPT-5, and 0.053 for GPT-5-Mini (Table SI3b). GPT-5-Mini was well calibrated across prompts, with ECE values below 0.10, consistent with accepted thresholds for image-classification tasks [31]. This improved calibration was also reflected in the high-confidence analyses.
2. High-confidence triage
  We implemented a post-hoc selective-classification layer with a reject option, Abstaining when the malignant probability $\:{p}_{\mathrm{Mal}}\:$fell between two decision thresholds ($\:{\tau\:}_{\mathrm{B}\mathrm{e}\mathrm{n}}<{p}_{\mathrm{Mal}}<{\tau\:}_{\mathrm{M}\mathrm{a}\mathrm{l}}$) [14, 21]. Thresholds were selected at the loss-function optimum and yielded near perfect accuracies (Fig. 2 & Fig.SI3). Using this method, the diagnostic performance was highest for GPT-5 with P-3: 98.1% accuracy (95% CI: 97.0–98.8), 94.6% specificity (95% CI: 90.5–96.9), and 99.1% sensitivity (95% CI: 98.1–99.6), F1 of 98.8% at 62.0% (889/1433) image coverage (CVG). In contrast, GPT-5-Mini with P-1 provided more robust CVG at 84.3%(1208/1433), with 94.5% accuracy (95% CI: 93.1–95.7), 88.5% specificity (95% CI: 85.0–91.2),97.6% Sensitivity (95% CI: 96.3–98.5), and F1 of 96.0% (Table S4).
3. Modality subgroups
  The diagnostic performance of NBI image classification task was highest for GPT-5-Mini with P-1: 84.4% accuracy (95% CI: 80.1–88.0), 77.7% specificity (95% CI: 69.1–84.4), 88.0% sensitivity (95% CI: 82.9–91.8), and F1 of 88.0%. (Table S5). Pearson χ² test found no significant accuracy difference across 3 Prompts (P-1 vs. P-2 vs. P-3) with GPT5.2, GPT5, GPT5-Mini, and GPT-5-Nano (*P* = 0.90, 0.74, 0.15 and 0.83 respectively). NBI vs. WLI Performance: For GPT-5-Mini, WLI outperformed NBI across all three prompts: the WLI–NBI accuracy differences, of GPT-5-Mini, were 4.4% (*P* = 0.097) with P-1, 10.1% (*P* < 0.001) with P-2, and 7.3% (*P* = 0.002) with P-3.

## Discussion

Cystoscopic assessment remains a central clinical decision point in BC diagnosis and surveillance because interpretation is visual, operator dependent, and pivotal for subsequent diagnostic and therapeutic decisions [5, 34]. This limitation is clinically important: cystoscopy can miss lesions, particularly flat lesions and carcinoma in situ, and the quality of tumor detection and resection influences recurrence risk [34]. WLI cystoscopy is still the everyday standard, but guidelines acknowledge that lesions may be missed and that enhanced visualization technologies were developed to address this gap [35].

In this context, the present work supports the role of MLLMs as clinically practical second readers: not as replacements for urologists, but as systems that can flag suspicious frames, structure visual attention, and provide consistent feedback during cystoscopy or training [21]. The main finding of this study is that PE improved the diagnostic behavior of off-the-shelf MLLMs on cystoscopic images. Real-time deep-learning cystoscopy systems have shown feasibility; however, they typically depend on purpose-built architectures, labeled training data, curated training pipelines, and site-specific validation. In contrast, MLLMs can be adapted through instructions alone. Here, we establish a simpler and faster complementary path: an instruction-driven layer that improves performance without model retraining. GPT-5 with P-3 was the strongest full-classification configuration, achieving 94.1% specificity and sensitivity of 82.5%. Importantly, across all tested models, P-3 produced the most favorable calibration profile, with lower BRS and improved ECE, indicating that the benefit extended beyond correct classification to more reliable probability estimates - an essential feature for a secondary reader.

Another significant finding is the performance of high-confidence triage. When the model abstained from low-confidence images, GPT-5-Mini with P-3 achieved 98.1% accuracy at 62.0% coverage, with 99.1% sensitivity and 94.6% specificity reaching state-of-the-art performance [36]. As a decision-support tool, cystoscopy AI does not need to classify every frame to be useful. The observed relationship between increasing abstention and improving accuracy supports a safety-oriented deployment strategy: a high-confidence assistant can defer ambiguous images to clinician judgment. This work also establishes a system in which image-quality problems, such as blur, glare, or poor focus, can be handled implicitly by rejecting uncertain frames rather than forcing a diagnosis [14]. Despite the encouraging performance demonstrated by the evaluated models, MLLM should not be implemented indiscriminately, as illustrated by GPT-5-Nano’s preference for the degenerate abstain-all solution. This finding highlights the need for guided model selection, continued task-specific development, and careful attention to potential inaccuracies and model-specific limitations.

The modality findings should be interpreted cautiously. Although NBI can improve lesion detection in selected clinical settings, our results did not demonstrate superior malignant detection with NBI [6]. This does not necessarily indicate that GPT-5 failed to recognize the same vascular or microtextural features used by human observers, as the model may rely on different and currently inaccessible visual representations. The findings may instead reflect limited representation of NBI images during GPT-5’s training corpus, differences in image quality or case complexity. Accordingly, the strong performance observed with WLI should not be interpreted as evidence that AI can replace NBI. Rather, it supports further controlled studies comparing WLI and NBI while investigating which image features contribute to model predictions.

A third positive implication is computational and operational (Table SI6). Because the intervention is prompt-based rather than model-training-based, it may be suitable for lower-compute workflows, including external processors or edge devices connected to endoscopy systems. Edge-AI endoscopy studies show that real-time diagnostic assistance can be implemented locally, reducing dependence on centralized infrastructure [37]. This matters for smaller hospitals and geographically remote centers, where specialist availability, infrastructure, and budget may limit access to advanced platforms. Rural and underserved patients face barriers to timely urologic care, and low-resource AI deployment is constrained by infrastructure, connectivity, data quality, and local capacity [38]. A prompt-optimized lightweight MLLM workflow could therefore be valuable if implemented on affordable hardware or through secure cloud/edge hybrid systems.

Finally, cystoscopy may serve as a model for a broader urologic and surgical transition. Image and video analysis are increasingly relevant in robotic surgery, endourology, laparoscopy, and surgical training, where AI can support anatomy recognition, phase detection, skill assessment, and real-time guidance [10, 12, 39]. The present work suggests that precise computational framing, here through PE may become an important determinant of performance across visual surgical AI systems.

### Limitations and future perspectives

This was a retrospective, image-based evaluation, whereas real-world cystoscopy is video-driven and temporally contextual. The study used a single public dataset comprising only 23 patients; therefore, external validation across diverse patient populations is essential to quantify generalizability. Future studies should integrate relevant clinical variables, including risk factors, presenting history, and findings from prior cystoscopy or transurethral resection, to determine whether MLLMs can improve clinical decision support and provide meaningful clinical value.

## Conclusion

In this retrospective evaluation of MLLM we demonstrated promising performance for bladder lesion classification. Prompt engineering modestly improved diagnostic performance and had a consistent effect on calibration, suggesting that structured task framing improve the reliability of model probability outputs. High-confidence triage using an abstention strategy achieved high accuracy (98.1%) among retained images while deferring uncertain cases. These findings highlight calibration and abstention as important components of safety-oriented AI evaluation. Overall, this work supports the feasibility of MLLMs as high-fidelity foundation models for cystoscopy assessment.

## Supplementary Information

Below is the link to the electronic supplementary material.

Supplementary Material 1

## Data Availability

No datasets were generated or analysed during the current study. Code and prompts are available from the corresponding author upon reasonable request.
