# Supplementary material for: Multimodal large language models for bladder tumor detection in cystoscopy: a retrospective benchmarking study
Source: World J Urol. 2026 Sep 17;44(1):670. doi: 10.1007/s00345-026-06761-y (PMC13585802; doi:10.1007/s00345-026-06761-y)
Supplement: Supplementary file 1 [file 345_2026_6761_MOESM1_ESM.docx]

Supplementary Information file

**Multimodal Large Language Models for Bladder Tumor Detection in Cystoscopy: A Retrospective Benchmarking Study**

**AI models and prompting strategies**

We evaluated four OpenAI multimodal large language models (MLLMs): GPT-5.2, GPT-5, GPT-5-Mini, and GPT-5-Nano. The specific API versions used were gpt-5.2-2025-12-11, gpt-5-2025-08-07, gpt-5-mini-2025-08-07, and gpt-5-nano-2025-08-07, respectively. All model evaluations were performed programmatically in Python version 3.12 within Google Colab using the official OpenAI Python SDK. Requests were submitted through the OpenAI Chat Completions API endpoint (https://api.openai.com/v1/chat/completions) using the client.chat.completions.create() method.. Images were encoded in base64 before submission. No model was fine-tuned.

Each image was evaluated under 12 independent inference conditions, corresponding to four models and three prompt configurations. This yielded 20,808 total model runs across 1,734 images. For each image, the model generated a four-class probability vector over non-suspicious tissue (NST), no-tumor lesion (NTL), low-grade carcinoma (LG), and high-grade carcinoma (HG). Outputs were constrained using an explicit JSON schema, requiring class probabilities to sum to 100%.

Each model was tested under three prompt configurations representing increasing levels of prompt-engineering complexity.

**Prompt selection and optimized prompt design**

The optimized prompt, P-3, incorporated several modifications intended to improve diagnostic reliability and reduce false-negative malignant classifications.

First, the system prompt included an explicit diagnostic objective, emphasizing conservative classification and minimization of missed malignancy. Making the optimization target explicit was intended to reduce output drift and inconsistent decision-making.

Second, the user prompt incorporated calibration instructions. Since the task required probability estimates, the model was instructed to assign high confidence only when classic visual hallmarks were present. This was intended to reduce overconfident benign predictions, which are particularly problematic in screening-style applications where negative predictive value is clinically important.

Third, the prompt included a lightweight self-check step. The model was instructed to briefly reconsider whether malignant features or image-quality limitations could support an alternative classification before finalizing the probability distribution. This was implemented without requiring verbose reasoning or additional free-text output.

Fourth, a limited guardrail was added. If malignancy could not be confidently excluded because of suspicious features or limited visibility, the model was instructed not to assign an overconfident benign probability distribution. This rule was designed to improve negative predictive value, with the expected trade-off of increased false-positive classifications.

**Few-shot prompt configuration**

An additional optimized few-shot prompt configuration, P-4, was also explored but was not included in the primary analysis because it did not improve classification accuracy. In this configuration, P-3 was augmented with a small set of labeled image-output examples, providing in-context demonstrations of the desired probability distributions for representative cases from each class.

**Dataset**

Two classes were considered malignant: low-grade carcinoma and high-grade carcinoma (LG ∪ HG). Two classes were considered benign: no-tumor lesion and non-suspicious tissue (NTL ∪ NST).

The dataset included both white-light imaging (WLI) and narrow-band imaging (NBI) frames. In total, 1,433 WLI images and 321 NBI images were included, as shown in Table S1. The dataset annotation file provided the filename, imaging modality, and class label for each image. Image dimensions were either 300 × 300 pixels or 350 × 250 pixels, with an average file size of 139 KB.

All frames were de-identified and renamed using random integer identifiers to prevent filename-based model leakage. WLI images were assigned identifiers from 1 to 1,433, and NBI images were assigned identifiers from 2,001 to 2,321.

**Computational efficiency**

Computational efficiency was assessed for each model by measuring the average inference time and cost per 1000 images under each evaluated prompting condition, as reported in Supplementary Table S6. Differences among prompting strategies were minimal, with variations of less than 5%. The total cost of the project, including preliminary prompt experimentation conducted before the final runs, was less than $20 ($19.63). The final evaluations using P-1, P-2, and P-3 cost $13.77, of which approximately 64% was attributable to GPT-5, 15% to GPT-5.2, 14% to GPT-5-Mini, and 6% to GPT-5-Nano.

**Model confidence assessment**

Model confidence and calibration were assessed using expected calibration error (ECE). We used an ECE sweep with 12 equal-mass bins, adapted to the available sample size, and calculated ECE separately for each model-prompt combination.

**Preprocessing and quality-control analysis**

A preprocessing and image-quality-control pipeline was initially developed and tested but was not used in the final analysis. The pipeline was ultimately abandoned to evaluate a more robust end-to-end system and to test whether high-confidence classification with abstention could handle blurry or low-quality images without explicit image exclusion. To identify motion blur and defocus, two no-reference sharpness metrics were calculated for each image. The first was variance of Laplacian (VoL), computed using the two-dimensional discrete Laplacian kernel:

A1. $\mathcal{L}_{xy}=\left| \begin{matrix} 0 & -1 & 0 \\ -1 & 4 & -1 \\ 0 & -1 & 0 \end{matrix} \right|$

Before calculating VoL and FFT-based sharpness, the bladder mucosa region was delineated using edge-based segmentation. The surrounding black background was masked so that it did not contribute to either sharpness metric, as shown in Figure S2.

Images below the fifth-percentile quality threshold were excluded in this exploratory analysis. This removed 88 images, corresponding to approximately 5% of the dataset, leaving 1,666 images for modeling. Because this preprocessing strategy was not used in the final analysis, these results are reported only as an exploratory quality-control experiment.

**Supplementary Tables and figures

Table SI1: Bladder Tissue Types - Patient Cases and Images**

| **Tissue type** | **No. of patient cases** | **No. of images** | | |
| --- | --- | --- | --- | --- |
|  |  | **WLI** | **NBI** | **Total** |
| **HG** | **8** | **405** | **64** | **469** |
| **LG** | **9** | **503** | **145** | **648** |
| **NST** | **5** | **429** | **75** | **504** |
| **NTL** | **5** | **97** | **37** | **134** |
| **Total** | **23*** | **1433** | **321** | **1754** |

**Table SI1: *The total number of patient cases does not correspond to the sum of the second column since some of the patients had more than one type of lesion.**

**Table SI2. Diagnostic statistics by model × prompt classification of WLI images.**

| **Model** | **Prompt** | **Acc % (95% CI)** | **Spec%  (95% CI)** | **Sens %  (95% CI)** | **NPV %  (95% CI)** | **PPV % (95% CI)** | **F1 %** |
| --- | --- | --- | --- | --- | --- | --- | --- |
| GPT-5.2 | P-1 | 74.4 (72.1-76.6) | 96.8 (94.9-98.0) | 61.4 (58.2-64.5) | 59.3 (55.9-62.5) | 97.0 (95.3-98.1) | 75.2 |
|  | P-2 | 72.8 (70.4-75.0) | 97.1 (95.3-98.3) | 58.7 (55.4-61.8) | 57.7 (54.4-60.9) | 97.3 (95.5-98.3) | 73.2 |
|  | P-3 | 75.4 (73.1-77.5) | 96.4 (94.4-97.7) | 63.2 (60.0-66.3) | 60.3 (56.9-65.3) | 96.8 (95.0-97.9) | 76.5 |
| GPT-5 | P-1 | 85.5 (83.6-87.2) | 94.3 (92.0-96.0) | 80.4 (77.7-82.8) | 73.6(70.1-76.8) | 96.0 (94.4-97.2) | 88.7 |
|  | P-2 | 86.4 (84.5-88.1) | 95.6 (93.5-97.1) | 81.0 (78.4-83.5) | 74.5 (71.1-77.7) | 97.0 (95.5-98.0) | 88.3 |
|  | P-3 | 86.7 (84.9-88.4) | 94.1 (91.8-95.8) | 82.5 (79.9-84.8) | 75.7 (72.3-78.8) | 96.0 (94.4-97.2) | 88.7 |
| GPT-5-Mini | P-1 | 88.8 (87.0-90.3) | 89.0 (86.0-91.4) | 88.6 (86.4-90.5) | 82.0 (78.6-84.9) | 93.3 (91.4-94.8) | 90.9 |
|  | P-2 | 88.6 (86.9-90.2) | 90.7 (87.9-92.9) | 87.4 (85.1-89.4) | 80.7 (77.3-83.7) | 94.2 (92.4-95.6) | 90.7 |
|  | P-3 | **89.2** (87.5-90.7) | 88.4 (85.4-90.9) | **89.6** (87.5-91.5) | **83.2** (79.9-86.1) | 93.0 (91.1-94.5) | **91.3** |
| GPT-5-Nano | P-1 | 45.6 (43.1-48.2) | 91.1 (88.3-93.2) | 19.3 (16.9-22.0) | 39.6 (36.8-42.3) | 78.8 (73.0-83.7) | 31.0 |
|  | P-2 | 48.0 (45.4-50.6) | 89.2 (86.2-91.5) | 24.1 (21.5-27.0) | 40.5 (37.7-43.4) | 79.3 (74.2-83.7) | 37.0 |
|  | P-3 | 50.6 (48.0-53.2) | 89.4 (86.4-91.7) | 28.1 (25.3-31.1) | 41.9 (39.0-44.8) | 82.0 (77.3-85.9) | 41.9 |

**Table SI2b. WLI - Pairwise McNemar Comparisons of Accuracy Between Models and Prompting Strategies With Holm Adjustment.**

| **Comparison family** | **Accuracy Comparison** | **Accuracy difference, percentage points** | **Unadjusted P (McNemar Comparisons)** | **Holm-adjusted P** |
| --- | --- | --- | --- | --- |
| Between models | GPT-5-Mini vs GPT-5, P-1 | 3.3+ | 0.0004 | **0.0011** |
| Between models | GPT-5-Mini vs GPT-5, P-2 | 2.2+ | 0.0092 | **0.0113** |
| Between models | GPT-5-Mini vs GPT-5, P-3 | 2.5+ | 0.0053 | **0.0105** |
| Within models | GPT-5.2, P-3 vs P-1 | +1.0 | 0.2392 | 0.9568 |
| Within models | GPT-5.2, P-3 vs P-2 | +2.6 | 0.0008 | **0.0055** |
| Within models | GPT-5, P-3 vs P-1 | +1.2 | 0.0763 | 0.3816 |
| Within models | GPT-5, P-3 vs P-2 | +0.3 | 0.6251 | 1.000 |
| Within models | GPT-5-Mini, P-3 vs P-1 | +0.4 | 0.5443 | 1.000 |
| Within models | GPT-5-Mini, P-3 vs P-2 | +0.6 | 0.3496 | 1.000 |
| Within models | GPT-5-Nano, P-3 vs P-1 | +5.0 | **<0.0001** | **<0.0001** |
| Within models | GPT-5-Nano, P-3 vs P-2 | +2.6 | 0.0367 | 0.2203 |

**Table SI3. Model confidence assessment by model × prompt classification of WLI images.**

| **Model** | **Prompt** | **BRS** | **ECE_sweep_** |
| --- | --- | --- | --- |
| **GPT-5.2** | **P-1** | **0.205** | **0.259** |
|  | **P-2** | **0.220** | **0.270** |
|  | **P-3** | **0.167** | **0.194** |
| **GPT-5** | **P-1** | **0.116** | **0.128** |
|  | **P-2** | **0.113** | **0.124** |
|  | **P-3** | **0.095** | **0.092** |
| **GPT-5-Mini** | **P-1** | **0.090** | **0.063** |
|  | **P-2** | **0.094** | **0.075** |
|  | **P-3** | **0.084** | **0.053** |
| **GPT-5-Nano** | **P-1** | **0.242** | **0.358** |
|  | **P-2** | **0.255** | **0.365** |
|  | **P-3** | **0.238** | **0.370** |

Lower Brier score (BRS) and expected calibration error ( ECE) indicate better calibration. Overall, Prompt 3 (P-3) showed the most favorable calibration profile, producing the lowest values in most models, especially GPT-5 and GPT-5-Mini

**Table SI3b. Two-sided P Comparisons of Brier Score Between Models and Prompting Strategies**

| **Model** | **Comparison** | **Difference** | **P value** |
| --- | --- | --- | --- |
| **GPT-5.2** | **P3 vs P2** | **-0.053** | **<0.001** |
| **GPT-5.2** | **P3 vs P1** | **-0.038** | **<0.001** |
| **GPT-5** | **P3 vs P2** | **-0.018** | **<0.001** |
| **GPT-5** | **P3 vs P1** | **-0.021** | **<0.001** |
| **GPT-5-Mini** | **P3 vs P2** | **-0.010** | **0.002** |
| **GPT-5-Mini** | **P3 vs P1** | **-0.006** | **0.077** |
| **GPT-5-Nano** | **P3 vs P2** | **-0.017** | **0.005** |
| **GPT-5-Nano** | **P3 vs P1** | **-0.004** | **0.572** |

**Table SI4: diagnostic statistics by model × prompt classification of WLI images using High-confidence Classification**

| **Model** | **Prompt** | **Acc % (95% CI)** | **Spec%  (95% CI)** | **Sens %  (95% CI)** | **NPV %  (95% CI)** | **PPV % (95% CI)** | **F1 %** | **CVG%** | $\tau_{\mathrm{Mal}}$ | $\tau_{Ben}$ |
| --- | --- | --- | --- | --- | --- | --- | --- | --- | --- | --- |
| GPT-5.2 | P-1 | 97.1 (95.5-98.1) | 89.0 (82.9-93.1) | 99.3 (98.1-99.7) | 97.0 (92.6-98.8) | 97.1 (95.3-98.2) | 98.2 | 47.7 | 56 | 1 |
|  | P-2 | 97.2 (95.6-98.2) | **96.3** (92.9-98.1) | 97.6 (95.7-98.7) | 95.4 (91.8-97.5) | 98.1 (96.3-99.0) | 97.9 | 44.7 | 75 | 1 |
|  | P-3 | 97.1 (95.5-98.1) | 92.3 (87.4-95.3) | 98.7 (97.3-99.4) | 96.0 (91.9-98.0) | 97.4 (95.7-98.4) | 98.0 | 49.7 | 62 | 7 |
| GPT-5 | P-1 | 96.2 (94.8-97.3) | 81.8 (75.2-87.0) | **99.5** (98.6-99.8) | 97.2 (92.8-98.9) | 96.0 (94.4-97.2) | 97.7 | 62.6 | 55 | 3 |
|  | P-2 | 97.9 (96.7-98.6) | 93.2 (88.9-95.9) | **99.3** (98.3-99.7) | **97.5** (94.2-98.9) | 98.0 (96.6-98.8) | 98.6 | 61.8 | 85 | 4 |
|  | P-3 | **98.1** (97.0-98.8) | **94.6** (90.5-96.9) | **99.1** (98.1-99.6) | 97.0 (93.5-98.6) | **98.4** (97.2-99.1) | **98.8** | 62.0 | 85 | 5 |
| GPT-5-Mini | P-1 | 94.5 (93.1-95.7) | **88.5** (85.0-91.2) | 97.6 (96.3-98.5) | 95.0 (92.3-96.8) | 94.3 (92.5-95.7) | 96.0 | **84.3** | 71 | 9 |
|  | P-2 | 95.7 (94.2-96.8) | 83.9 (78.3-88.3) | 99.0 (98.0-99.5) | 96.1 (92.1-98.1) | 95.6 (93.8-96.8) | 97.3 | 64.4 | 83 | 4 |
|  | P-3 | **96.3** (94.8-97.4) | 74.8 (66.1-81.8) | **99.7** (99.0-99.9) | **97.7** (92.1-99.4) | **96.1** (94.5-97.3) | 97.9 | 58.9 | 81 | 4 |
| GPT-5- Nano* | P-1 | NA | NA | NA | NA | NA | NA | NA | NA | NA |
|  | P-2 | NA | NA | NA | NA | NA | NA | NA | NA | NA |
|  | P-3 | NA | NA | NA | NA | NA | NA | NA | NA | NA |

*GPT-5- Nano’s loss function favored the trivial solution, to abstain all images, for any $\tau_{\mathrm{Mal}}, \tau_{Ben}$. NA - Not Available

**Table S5. diagnostic statistics by model × prompt classification of NBI images:**

| **Model** | **Prompt** | **Acc % (95% CI)** | **Spec%  (95% CI)** | **Sens %  (95% CI)** | **NPV %  (95% CI)** | **PPV % (95% CI)** | **F1 %** |
| --- | --- | --- | --- | --- | --- | --- | --- |
| GPT-5.2 | P-1 | 80.4 (75.7-84.3) | **88.4** (81.1-93.1) | 76.1 (69.9-81.4) | 66.4 (58.5-73.5) | **92.4** (87.5-95.5) | 83.5 |
|  | P-2 | 80.4 (75.7-84.3) | 86.6 (79.1-91.7) | 77.0 (70.9-82.2) | 66.9 (58.9-74.0) | 91.5 (86.4-94.8) | 83.6 |
|  | P-3 | 81.6 (77.0-85.5) | 83.0 (75.0-88.9) | 80.9 (75.0-85.6) | 69.9 (61.7-77.1) | 89.9 (84.8-93.4) | 85.1 |
| GPT-5 | P-1 | 81.6 (77.0-85.5) | 84.8 (77.0-90.3) | 79.9 (74.0-84.8) | 69.3(61.2-76.4) | 90.8 (85.7-94.2) | 85.0 |
|  | P-2 | 79.8 (75.0-83.8) | 83.0 (75.0-88.9) | 78.0 (71.9-83.1) | 66.9 (58.7-74.2) | 89.6 (84.3-93.2) | 83.4 |
|  | P-3 | 81.9 (77.4-85.8) | 82.1 (74.0-88.1) | 81.8 (76.0-86.5) | 70.8 (62.4-77.9) | 89.6 (84.4-93.1) | 85.5 |
| GPT-5-Mini | P-1 | **84.4** (80.1-88.0) | 77.7 (69.1-84.4) | **88.0** (82.9-91.8) | **77.7** (69.1-84.4) | 88.0 (82.9-91.8) | **88.0** |
|  | P-2 | 78.5 (73.7-82.6) | 72.3 (63.5-79.8) | 81.8 (76.0-86.5) | 68.1 (59.2-75.8) | 84.7 (79.0-89.0) | 83.2 |
|  | P-3 | 81.9 (77.4-85.8) | 75.0 (66.2-82.1) | 85.6 (80.2-89.8) | 73.7 (64.9-80.9) | 86.5 (81.1-90.5) | 86.1 |
| GPT-5- Nano | P-1 | 46.1 (40.7-51.6) | 82.1 (74.0-88.1) | 26.8 (21.2-33.2) | 37.6 (31.7-43.8) | 73.7 (62.8-82.3) | 39.3 |
|  | P-2 | 48.3 (42.9-53.7) | 80.4 (72.0-86.7) | 31.1 (25.2-37.7) | 38.5 (32.5-44.8) | 74.7 (64.7-82.7) | 43.9 |
|  | P-3 | 48.0 (42.6-53.4) | 77.7 (69.1-84.4) | 32.1 (26.1-38.7) | 38.0 (32.0-44.4) | 72.8 (63.0-80.9) | 44.5 |

| **Model** | **GPT5 Nano** | **GPT5 Mini** | **GPT 5** | **GPT5.2** |
| --- | --- | --- | --- | --- |
| **Runtime [Sec/Image]** | 1.08 | 2.41 | 4.25 | 0.93 |
| **Cost [$/1000 Images]** | 0.16$ | 0.40$ | 1.81$ | 0.42$ |

Table S6. Average runtime and cost per image across 4 models.

**Table S6.** Computational efficiency of different models

**Figure SI1: Study Overview**

Figure 1: A total of 1,754 images were captured from cystoscopy videos from 23 patients. Images were reviewed by expert urologists and then labelled with pathological information. Accuracy (Acc), sensitivity (Sens), specificity (Spec), positive predictive value (PPV), negative predictive value (NPV), and F1-score (F1).

**Figure SI2: Post-hoc selective classification layer**

**Figure 2.** High-confidence triage with selective classification. Frozen model outputs are classified as benign or malignant only when pMal crosses predefined thresholds; intermediate-probability cases are abstained and deferred to standard-of-care human review, reducing automated coverage in exchange for higher reliability.

**Figure SI3: 3D Loss Function**$\left( \boldsymbol{\tau}_{\boldsymbol{Mal}} , \boldsymbol{\tau}_{\boldsymbol{Ben}} \right)$

**Figure S3:** Loss Function$\left( \tau_{Mal} , \tau_{Ben} \right)$ was recorded, using discrete sweep over two-sided threshold updating thresholds in 1% increments.

**Figure SI4: Accuracy by model × prompt classification of WLI images**

**Figure SI4.** Accuracy across models and prompt. GPT-5-Mini achieved the highest WLI classification accuracy across all prompts, followed by GPT-5. P3 generally showed the best performance, especially in GPT-5-Nano, where accuracy improved from 45.6% with P1 to 50.6% with P3.

**Figure SI5: Image edge-based segmentation**

**Fig. SI5**

**Figure SI5**: Image edge-based segmentation

Bladder mucosa was circled in green using edge-based segmentation, masking black background for VoL and FTT calculation. Left - raw images. Right - circled bladder mucosa. Up - image filtered out. Bottom - image filtered in.
